# Polymorphisms of *TLR2, TLR4* and *TOLLIP* and tuberculosis in two independent studies

**DOI:** 10.1042/BSR20193141

**Published:** 2020-08-03

**Authors:** Shouquan Wu, Xiangmin Liu, Ling Chen, Yu Wang, Miaomiao Zhang, Minggui Wang, Jian-Qing He

**Affiliations:** Department of Respiratory and Critical Care Medicine, West China Hospital, Sichuan University, Chengdu, Sichuan, China

**Keywords:** susceptibility, toll interacting protein, toll-like receptor 2, 4, tuberculosis

## Abstract

Genetic polymorphisms for tuberculosis (TB) susceptibility have been researched by some studies, but few have studied multiple innate immunity genes associated with TB. Evidence suggests that the toll-like receptor 2, 4 (TLR2, TLR4) and toll interacting protein (*TOLLIP*) may be associated with TB susceptibility. In this self-validated study, we explored the association between common single nucleotide polymorphisms (SNPs) of *TLR2, TLR4* and *TOLLIP* in the Chinese Han and Tibetan populations. A SNPscan™ method was used to genotype SNPs in the three genes. Multiple logistic regression adjusted by sex and age was used to detect the association between SNPs and TB. In *TLR2*, rs1898830 was associated with decreased risk against TB in the Chinese Han population, which was validated in the Tibetan population. In *TLR4*, rs11536889 was a protective factor for TB in the Tibetan population, but not in the Han population. Additionally, in the Tibetan population, we also found that the frequency of genotypes of *TOLLIP* rs11536889 differs significantly between TB patients and controls. We found rs1898830 in *TLR2* was associated with TB susceptibility in both Chinese Han and Tibetan populations while rs11536889 in *TLR4* and rs3750920 in *TOLLIP* were protective factors against TB in the Tibetan population.

## Introduction

Tuberculosis (TB) remains a serious global health concern. In 2017, World Health Organization (WHO) estimated that approximately 10.4 million new TB cases were diagnosed and 1.7 million died from it, majorly in developing countries [1]. Almost one-third of the population is infected with *Mycobacterium tuberculosis* (*M. TB*), however, only 10% of them develop active TB, indicating that there are discrepancies among individuals in the susceptibility to TB development. In addition to environmental factors, host genetic factors also play an important role in TB vulnerability [2]. Studies of *M. TB* infections in both humans and mice have reported several potential TB causal genes, including genes related to toll-like receptor (TLR) signaling [3].

The innate immune system activated by pathogenic bacteria acts as the first-line host defense mechanism, which recognizes and phagocytizes the invading pathogen [4]. The TLRs are pattern recognition receptors participants in this innate immune recognition of pathogens and stimulate the response to adaptive immune. TLR are transmembrane proteins comprising ten receptors (TLR1–10), with functions of binding ligands on cell surfaces and in the cytosolic compartment [5]. *M. TB* is initially discerned by TLR2 and TLR4, which subsequently interact with toll interacting protein (TOLLIP) to activate macrophages [6–8].

Because TLRs are involved in the activation of the inflammatory cytokine signaling pathways and the response to adaptive immune, they have become biologically causal genes in studies of TB [9,10]. Our published studies demonstrated that *TLR1* and *TLR9* were associated with TB [11,12]. Other studies from various populations reported that different TLR pathway genes, including *TLR2* [13], *TLR4* [13] and *TOLLIP* [6], activate the cellular immune response and might affect individual susceptibility to TB. Although numerous studies have been conducted to research the association between single nucleotide polymorphisms (SNPs) of *TLR2, TLR4*, and *TOLLIP* and TB susceptibility, the results have not been replicated among different populations.

In the present study, we used alleles, genotypes and different genetic models to evaluate whether the functional SNPs in *TLR2, TLR4* and *TOLLIP* could contribute to TB susceptibility. We performed this genotyping analysis in two independent studies in TB patients and TB-negative controls.

## Materials and methods

### Cases and controls

In the initial study, we recruited a total of 636 TB cases and 608 TB-negative controls from West China Hospital of Sichuan University. To validate the results of the initial study, we performed an independent study on 613 TB patients and 603 healthy individuals from the People’s Hospital of Aba Tibetan Autonomous Prefecture. The diagnosis of TB was established according to the WHO criteria. Briefly, TB was diagnosed with a combination of radiological evidence, clinical symptoms, bacteriological investigations and response to anti-TB therapy. As control groups, we recruited individuals based on the absence of any evidence or history of TB. All participants in both initial and validated studies with immune disorders, diabetes, immune-related diseases, and HIV infection were excluded. Also, they have no blood relationship. Written informed consent was required for each participant, and 2–5 ml peripheral blood was collected in EDTA tubes and stored at −80°C until DNA extraction and genotyping. The Ethics Committees of the West China Hospital of Sichuan University and the People’s Hospital of the Aba Tibetan Autonomous Prefecture provided approval for the present study.

### SNPs selection and genotyping

Genomic DNA was obtained from the whole blood sample according to the manufacturer’s instructions (Axygen Scientific Inc, Union City, CA, U.S.A.). Potentially functional SNPs were selected based on previously reported functional effects, and *in silico* functional prediction from the FuncPred (http://snpinfo.niehs.nih.gov/snpinfo/snpfunc.htm). Genotyping was done by the improved multiplex ligase detection reaction (iMLDR) (Cat#: G0104, Genesky Biotechnologies Inc., Shanghai, China) as described previously [14]. For quality control, 5% of the randomly selected subjects were repeated by iMLDR to confirm the genotyping results.

### Statistical analysis

Sex distribution between the cases and controls were calculated by using χ^2^ test. Fisher’s exact test was used to evaluate the Hardy–Weinberg equilibrium (HWE) of the control groups. Continuous variable (shown with mean ± SD) was calculated by Student’s *t* test. SHEsis online software platform (http://analysis.bio-x.cn) was used to calculate haplotype and linkage disequilibrium (LD) (using r^2^ as coefficients) between SNPs. The association between genotype/genetic model and TB was examined using a logistic regression analysis adjusted by sex and age. *P*-values <0.05 were considered to be statistically significant for all tests. All analyses were conducted by SPSS version 19 (IBM, Armonk, NY).

## Results

### Demographics of the participants and results of quality control

The clinical characteristics of the two studies are summarized in Table 1. A total of 636 TB cases and 608 TB-negative controls were recruited from the Chinese Han population for genetic analysis. There was no significant difference in either age or sex distributions between the cases (mean age: 36.8 ± 15.7 years, sex: 324 males and 312 females) and controls (mean age: 37.1 ± 15.7 years, sex: 302 males and 306 females). In the validated study, we enrolled 613 cases and 603 controls from Chinese Tibetan population. Also, the age and sex distributions in this cohort between the patients (mean age: 34.5 ± 14.5 years, sex: 327 males and 286 females) and controls (mean age: 34.6 ± 13.8 years, sex: 333 males and 270 females) were not significantly different. Briefly, the cases and controls were well matched for age and sex in the two studies.

**Table 1:** Demographic distribution of TB-negative controls and TB patients

Parameters	Cases	Controls	*P*-value
Han population	*n*=636	*n*=608	
Age, (years)*	36.8 ± 15.7	37.1 ± 15.7	0.677
Male, *n* (%)	324 (50.9%)	302 (49.7%)	0.654
Location of TB, *n* (PTB/EPTB)	276/360		
Acid-fast bacilli stain positive, *n* (positive/negative)	138/360		
Culture positive *n* (positive/negative)	32/126		
TB-DNA positive *n* (positive/negative)	122/133		
Tibetan population	*n*=613	*n*=603	
Age, (years)*	34.5 ± 14.5	34.6 ± 13.8	0.909
Male, *n* (%)	327 (53.3%)	333 (55.2%)	0.511

Abbreviations: EPTB, extra-pulmonary TB PTB, pulmonary TB; SD, standard deviation.

* Data are presented as mean ± SD.

### Polymorphisms of the three genes in the two studies

According to literature review and FuncPred, four SNPs (rs1898830, rs3804099, rs3804100 and rs5743708) in *TLR2*, four SNPs (rs10759932, rs12377632, rs11536889 and rs7873784) in *TLR4* and three SNPs (rs3750920, rs5743899 and rs5743867) in *TOLLIP* were retained for analysis (Table 2). None of the SNPs deviated from HWE.

**Table 2:** Basic information of all SNPs in our study

Gene/SNPs	Chromosome	Location	Functional consequence	MA	MAF	MA	MAF	HWE	
				Han		Tibetan		Han	Tibetan
*TLR2*									
rs1898830	4	154608453	intron1	G	0.406	G	0.461	0.839	0.456
rs3804099	4	154624656	synon_exon3	C	0.303	C	0.224	0.830	0.575
rs3804100	4	154625409	synon_exon3	C	0.260	C	0.204	0.985	0.963
rs5743708	4	154626317	nonsynon_exon3	A	0.000	A	0.000	-	-
*TLR4*									
rs10759932	9	120465144	5′Flanking	C	0.263	C	0.325	0.608	0.830
rs12377632	9	120472730	intron3	T	0.387	T	0.436	0.996	0.828
rs11536889	9	120478131	3′UTR_exon4	C	0.245	C	0.155	0.563	0.748
rs7873784	9	120478936	3′UTR_exon4	C	0.084	C	0.066	0.934	0.690
*TOLLIP*									
rs3750920	11	1309956	synon_exon4	T	0.309	T	0.285	0.751	0.100
rs5743899	11	1323564	intron1	C	0.374	C	0.365	0.824	0.870
rs5743867	11	1328351	intron1	G	0.365	G	0.356	0.652	0.797

Abbreviations: MA, minor allele; MAF, MA frequency.

**Figure 1:**
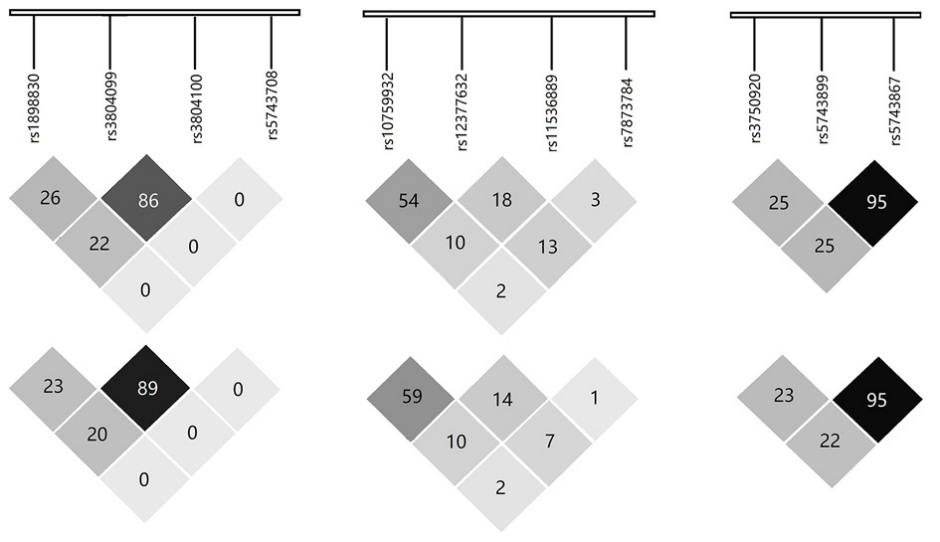
LD of *TLR2, TLR4* and *TOLLIP* gene polymorphisms in the both Han (above) and Tibetan (down) populations LD r^2^ values (range from 0 to 1) for all pairs of SNPs are presented as percentages. Shading from white to black indicates LD measured as r^2^ (range from 0 to 1).

The genotypic and allelic frequencies of the two studies are shown in Table 3; genetic models are shown in Table 4. In the initial Chinese Han population, the frequency of *TLR2* rs1898830 G allele was lower in TB patients than in controls (*P* = 0.035, odds ratio (OR) = 0.84, 95% confidence interval (CI): 0.72–0.99), indicating it was a protective factor against TB. We also found rs1898830 GG genotype was associated with decreased risk for TB development (*P*=0.042, OR = 0.72, 95% CI: 0.52–0.99). For rs5743708, all participants were detected to be monomorphic except for two patients with GA heterozygote. There was no statistical significance in allelic or genotypic frequencies between cases and controls for *TLR4* and *TOLLIP* in this cohort. Three haplotypes in *TLR2* showed a value of *P*<0.05, e.g., ACTG (*P*=0.025), ATTG (*P*=0.048) and GTTG (*P*=0.035) (Table 5). However, the sites rs3804099 and rs3804100 of TLR2 were in high LD, and rs5743899 and rs5743867 of *TOLLIP* also were in high LD (r^2^ > 0.8) (Figure 1).

**Table 3:** Genotype distribution of *TLR2, TLR4* and *TOLLIP* in the two populations

		Han population				Tibetan population			
Gene/SNPs		Case (%), *n*=636	Control (%), *n*=608	P^*^	OR^*^ (95% CI)	Case (%), *n*=613	Control (%), *n*=603	P^*^	OR^*^ (95% CI)
*TLR2*									
rs1898830	Genotype								
	AA	227 (25.9)	188 (31.1)			191 (31.2)	152 (25.2)		
	GA	297 (47.0)	292 (48.3)	0.193	0.85 (0.66–1.09)	279 (45.5)	316 (52.4)	0.009	0.70 (0.54–0.92)
	GG	108 (17.7)	125 (20.7)	0.042	0.72 (0.52–0.99)	143 (23.3)	135 (22.4)	0.307	0.85 (0.62–1.17)
	Allele								
	A	751 (59.4)	668 (55.2)			661 (53.9)	620 (51.4)		
	G	513 (40.6)	542 (44.8)	0.035	0.84 (0.72–0.99)	565 (46.1)	586 (48.6)	0.229	0.91 (0.77–1.06)
rs3804099	Genotype								
	TT	313 (49.5)	294 (48.6)			374 (61.0)	357 (59.3)		
	CT	255 (40.3)	260 (43.0)	0.505	0.92 (0.73–1.17)	203 (33.1)	219 (36.4)	0.291	0.88 (0.69–1.12)
	CC	64 (10.1)	51 (8.4)	0.457	1.17 (0.78–1.74)	36 (5.9)	26 (4.3)	0.302	1.32 (0.78–2.23)
	Allele								
	T	881 (69.7)	848 (70.1)			951 (77.6)	933 (77.5)		
	C	383 (30.3)	362 (29.9)	0.826	1.02 (0.86–1.21)	275 (22.4)	271 (22.5)	0.951	0.99 (0.82–1.20)
rs3804100	Genotype								
	TT	331 (52.6)	330 (54.6)			387 (63.1)	381 (63.2)		
	CT	247 (39.3)	234 (38.7)	0.654	1.06 (0.83–1.34)	196 (32.0)	198 (32.8)	0.804	0.97 (0.76–1.24)
	CC	51 (8.1)	40 (6.6)	0.241	1.30 (0.84–2.00)	30 (4.9)	24 (4.0)	0.501	1.21 (0.69–2.11)
	Allele								
	T	909 (72.3)	894 (74.0)			970 (79.1)	960 (79.6)		
	C	349 (27.7)	314 (26.0)	0.267	1.11 (0.93–1.32)	256 (20.9)	246 (20.4)	0.785	1.03 (0.84–1.25)
rs5743708	Genotype								
	GG	629 (99.7)	605 (1.0)			613 (1.0)	601 (99.7)		
	GA	2 (0.3)	0 (0.0)	-		0 (0.0)	2 (0.3)	-	
	AA	0 (0.0)	0 (0.0)	-		0 (0.0)	0 (0.0)	-	
	Allele								
	G	1260 (99.8)	1210 (1.0)			1226 (1.0)	1204 (99.8)		
	A	2 (0.2)	0 (0.0)	-		0 (0.0)	2 (0.2)	-	
*TLR4*									
rs10759932	Genotype								
	TT	347 (54.9)	322 (53.2)			256 (41.8)	278 (46.1)		
	CT	238 (37.7)	232 (38.3)	0.628	0.94 (0.74–1.20)	277 (45.2)	258 (42.8)	0.193	1.17 (0.92–1.49)
	CC	47 (7.4)	51 (8.4)	0.499	0.46 (0.57–1.32)	80 (13.1)	67 (11.1)	0.176	1.29 (0.89–1.86)
	Allele								
	T	932 (73.7)	876 (72.4)			789 (64.4)	814 (67.5)		
	C	332 (26.3)	334 (27.6)	0.433	0.93 (0.78–1.11)	437 (35.6)	392 (32.5)	0.098	1.15 (0.97–1.36)
rs12377632	Genotype								
	CC	232 (36.7)	227 (37.5)			178 (29.0)	188 (31.2)		
	CT	304 (48.1)	288 (47.6)	0.808	1.03 (0.81–1.31)	316 (51.5)	304 (50.4)	0.473	1.10 (0.85–1.42)
	TT	96 (15.2)	90 (14.9)	0.797	1.05 (0.74–1.47)	119 (19.4)	111 (18.4)	0.460	1.13 (0.81–1.58)
	Allele								
	C	768 (60.8)	742 (61.3)			672 (54.8)	680 (56.4)		
	T	496 (39.2)	468 (38.7)	0.785	1.02 (0.87–1.20)	554 (45.2)	526 (43.6)	0.429	1.07 (0.91–1.25)
rs11536889	Genotype								
	GG	361 (57.1)	339 (56.0)			436 (71.1)	387 (64.3)		
	GC	232 (36.7)	221 (36.5)	0.911	0.99 (0.78–1.25)	164 (26.8)	195 (32.4)	0.021	0.75 (0.58–0.96)
	CC	39 (6.2)	45 (7.4)	0.358	0.81 (0.51–1.27)	13 (2.1)	20 (3.3)	0.129	0.58 (0.28–1.18)
	Allele								
	G	954 (75.5)	899 (74.3)			1036 (84.5)	969 (80.5)		
	C	310 (24.5)	211 (25.7)	0.492	0.84 (0.78–1.13)	190 (15.5)	235 (19.5)	0.009	0.76 (0.61–0.93)
rs7873784	Genotype								
	GG	523 (82.8)	508 (84.0)			534 (87.1)	516 (85.6)		
	GC	105 (16.6)	92 (15.2)	0.498	1.11 (0.82–1.51)	77 (12.6)	82 (13.6)	0.571	0.91 (0.6–1.27)
	CC	4 (0.6)	5 (0.8)	0.701	0.77 (0.21–2.90)	2(0.3)	5(0.8)	0.280	0.40 (0.08–2.10)
	Allele								
	G	1151 (91.1)	1108 (91.6)			1145 (93.4)	1114 (92.4)		
	C	113 (8.9)	102 (8.4)	0.638	1.07 (0.81–1.42)	81 (6.6)	92 (7.6)	0.335	0.86 (0.63–1.17)
*TOLLIP*									
rs3750920	Genotype								
	CC	294 (46.5)	288 (47.6)			310 (50.6)	303 (50.2)		
	CT	286 (45.3)	253 (41.8)	0.406	1.11 (0.87–1.40)	256 (41.8)	233 (38.6)	0.556	1.07(0.85–1.36)
	TT	52 (8.2)	64 (10.6)	0.279	0.80 (0.54–1.20)	47 (7.7)	67 (11.1)	0.065	0.68 (0.45–1.02)
	Allele								
	C	874 (69.1)	829 (68.5)			876 (71.5)	839 (69.6)		
	T	390 (30.9)	381 (31.5)	0.711	0.97 (0.82–1.15)	350 (28.5)	367 (30.4)	0.302	0.91 (0.77–1.09)
rs5743899	Genotype								
	TT	248 (39.2)	241 (39.8)			249 (40.6)	241 (40.0)		
	CT	291 (46.0)	276 (45.6)	0.850	1.02 (0.80–1.30)	280 (45.7)	275 (45.7)	0.912	0.99 (0.77–1.26)
	CC	93 (14.7)	88 (14.5)	0.873	1.03 (0.73–1.45)	84 (13.7)	86 (14.3)	0.748	0.94 (0.97–1.40)
	Allele								
	T	787 (62.3)	758 (62.6)			778 (63.5)	757 (62.9)		
	C	477 (37.7)	452 (37.4)	0.834	1.02 (0.87–1.20)	448 (36.5)	447 (37.1)	0.764	0.98 (0.83–1.15)
rs5743867	Genotype								
	AA	254 (40.2)	249 (41.2)			256 (41.8)	249 (41.3)		
	GA	289 (45.7)	270 (44.6)	0.698	1.05 (0.8–1.34)	278 (45.4)	271 (44.9)	0.985	0.99 (0.78–1.27)
	GG	89 (14.1)	86 (14.2)	0.935	1.01 (0.72–1.43)	79 (12.9)	83 (13.8)	0.668	0.93 (0.65–1.32)
	Allele								
	A	797 (63.1)	768 (63.5)			790 (64.4)	769 (63.8)		
	G	467 (36.9)	442 (36.5)	0.817	1.02 (0.87–1.20)	436 (35.6)	437 (36.2)	0.726	0.97 (0.82–1.15)

* Adjusted by age and sex status.

**Table 4:** Association between genotype of *TLR2, TLR4* and *TOLLIP* and TB in the two populations

Gene/SNPs	Han population		Tibetan population	
	P^*^	OR^*^ (95% CI)	P^*^	OR^*^ (95% CI)
*TLR2*				
rs1898830				
Dominant model	0.074	0.81 (0.94–1.02)	0.023	0.75 (0.58–0.96)
Recessive model	0.106	0.79 (0.59–1.05)	0.680	1.06 (0.81–1.38)
rs3804099				
Dominant model	0.752	0.97 (0.77–1.21)	0.525	0.93 (0.74–1.69)
Recessive model	0.300	1.23 (0.83–1.81)	0.213	1.39 (0.83–2.34)
rs3804100				
Dominant model	0.437	1.09 (0.87–1.37)	0.993	0.99 (0.79–1.26)
Recessive model	0.243	1.29 (0.84–1.96)	0.436	1.25 (0.72–2.16)
rs5743708				
Dominant model	-		-	
Recessive model	-		-	
*TLR4*				
rs10759932				
Dominant model	0.522	0.93 (0.74–1.16)	0.119	1.20 (0.96–1.50)
Recessive model	0.521	0.87 (0.58–1.32)	0.304	1.20 (0.85–1.70)
rs12377632				
Dominant model	0.783	1.03 (0.82–1.30)	0.408	1.11 (0.87–1.42)
Recessive model	0.878	1.03 (0.75–1.40)	0.653	1.07 (0.80–1.42)
rs11536889				
Dominant model	0.697	0.96 (0.76–1.20)	0.011	0.73 (0.57–0.93)
Recessive model	0.365	0.81 (0.52–1.27)	0.204	0.63 (0.34–1.28)
rs7873784				
Dominant model	0.440	0.88 (0.63–1.22)	0.554	1.10 (0.81–1.48)
Recessive model	0.273	0.40 (0.77–2.07)	0.697	0.77 (0.21–2.88)
*TOLLIP*				
rs3750920				
Dominant model	0.720	1.04 (0.83–1.30)	0.901	0.99 (0.79–1.24)
Recessive model	0.149	0.75 (0.51–1.11)	0.040	0.66 (0.45–0.98)
rs5743899				
Dominant model	0.828	1.03 (0.82–1.29)	0.838	0.98 (0.78–1.23)
Recessive model	0.915	1.02 (0.74–1.40)	0.762	0.95 (0.69–1.32)
rs5743867				
Dominant model	0.730	1.04 (0.83–1.31)	0.866	0.98 (0.78–1.23)
Recessive model	0.965	0.99 (0.72–1.37)	0.649	0.93 (0.67–1.29)

* Adjusted by sex and age.

**Table 5:** Haplotype analyses in the two populations

	Han population				Tibetan population			
Gene/haplotype	Case (%), *n*=1264	Control (%), *n*=1210	*P*	OR (95% CI)	Case (%), *n*=1226	Control (%), *n*=1210	*P*	OR (95% CI)
TLR2								
ACCG	342.1 (27.1)	302.4 (25.0)	0.235	1.12 (0.93–1.34)	249.0 (20.3)	238.1 (19.8)	0.817	1.02 (0.84–1.25)
ACTG	27.4 (2.2)	44.7 (3.7)	0.025	0.58 (0.36–0.94)				
ATTG	380.3 (30.1)	321.0 (26.5)	0.048	1.19 (1.00–1.42)	393.0 (32.1)	351.9 (29.2)	0.165	1.13 (0.95–1.35)
GTTG	497.7 (39.4)	527.0 (43.6)	0.035	0.84 (0.72–0.99)	558.0 (45.5)	579.1 (48.1)	0.140	0.89 (0.75–1.04)
Other*	14.4 (0.01)	15.0 (1.2)			26.0 (2.1)	34.9 (2.9)		
TLR4								
CTGG	318.4 (25.2)	320.9 (26.5)	0.429	0.93 (0.78–1.11)	417.6 (34.1)	377.9 (31.4)	0.149	1.13 (0.96–1.34)
TCCG	297.9 (23.6)	304.6 (25.2)	0.335	0.91 (0.76–1.10)	188.4 (15.4)	222.9 (18.5)	0.040	0.80 (0.65–0.99)
TCGG	464.5 (36.8)	424.6 (35.1)	0.410	1.07 (0.91–1.26)	464.3 (37.9)	446.8 (37.1)	0.670	1.04 (0.88–1.22)
TTGC	108.3 (8.6)	93.0 (7.7)	0.433	1.12 (0.84–1.50)	70.2(5.7)	85.3 (7.1)	0.175	0.80 (0.58–1.11)
TTGG	54.4 (4.3)	44.6 (3.7)	0.441	1.17 (0.78–1.76)	59.6 (4.9)	47.9 (4.0)	0.286	1.24 (0.84–1.82)
Other*	20.5 (1.6)	22.3 (1.8)			25.9 (2.1)	23.2 (2.0)		
TOLLIP								
CCG	463.9 (36.7)	400.0 (36.4)	0.879	1.01 (0.86–1.19)	433.5 (35.4)	4345.0 (36.1)	0.705	0.97 (0.82–1.14)
CTA	402.9 (31.9)	379.6 (31.4)	0.803	1.02 (0.86–1.21)	433.8 (35.4)	392.1 (32.6)	0.137	1.14 (0.96–1.35)
TTA	384.1 (30.4)	376.4 (31.1)	0.682	0.97 (0.81–1.15)	343.2 (28.0)	362.9 (30.1)	0.250	0.90 (0.76–1.08)
Other*	13.1 (1.0)	14.1 (1.2)			15.6 (1.2)	14.1 (1.2)		

* Those with lowest frequency threshold (LFT) < 0.03 were pooled in this part.

In the validated Chinese Tibetan study, *TLR2* rs1898830 polymorphism was associated with decreased risk for TB, which was in accordance with the result of the initial study. Compared with AA genotype, both the GA (*P*=0.009, OR = 0.70, 95% CI: 0.54–0.92) and GG+GA (*P*=0.023, OR = 0.75, 95% CI: 0.58–0.96) were found to be protective factors against TB susceptibility. For *TLR4*, the frequencies of rs11536889 C allele (*P*=0.009, OR = 0.76, 95% CI: 0.61–0.93) and GC genotype (*P*=0.021, OR = 0.75, 95% CI: 0.58–0.96) were higher in controls as compared with TB cases. We also found the rs11536889 CC+GC to be a significant protective factor against TB under a dominant model (*P*=0.011, OR = 0.73, 95% CI: 0.57–0.93). For *TOLLIP*, only the rs3750920 site was found to be associated with the occurrence of TB in the recessive model (*P*=0.040, OR = 0.66, 95% CI: 0.45–0.98). Haplotype analysis revealed that *TLR4* TCCG haplotype was associated with TB (*P*=0.040, OR = 0.80, 95% CI: 0.65–0.99) (Table 5). High LD (r^2^ > 0.8) was found between the two *TLR2* loci (rs3804099 and rs3804100) and two *TOLLIP* loci (rs5743899 and rs5743867) (Figure 1).

## Discussion

Accumulated studies have demonstrated that *TLR2, TLR4* and *TOLLIP* are pivotal mediators of individual response to and etiology of various pathogens causing common human diseases, including TB. In this self-validating association study, we researched the impact of functional SNPs in *TLR2, TLR4* and *TOLLIP* and TB in two independent cohorts. Our findings revealed that *TLR2* polymorphism was associated with TB development in the Chinese Han population, which was validated in the Tibetan study. We also observed that TLR4 and TOLLIP genetic variants were protective factors against TB in the Tibetan population, but not in the Han population.

Although microbe exposure and environmental factors play important roles in TB development, there is convincing evidence regarding the causal gene of TB susceptibility [11,12,15,16]. TLRs family members are important to defend against *M. TB* infection. TLR2, as a family member of TLRs, could activate an immunoreaction against *M. TB* via recognizing *M. TB* components and shaping pro-inflammatory signals [17]. Abundant studies proposed that *TLR2* polymorphisms were involved in bacterial infections and various diseases [15,18–20]. In the present study, the allele or genotype distribution of rs1898830 strongly differed between TB cases and controls in the initial cohort and it was replicated in the Tibetan sample. One study has reported that rs1898830 GG was related to low *FOXP3, GITR* and *LAG3* expression in cord blood mononuclear cells and it also can reduce the T helper 2 cell and tumor necrosis factor secretion [21]. Combined with the above aspects, the *TLR2* rs1898830 polymorphism might be a causative factor for TB susceptibility. Previous studies have demonstrated that rs3804099, rs3804100 and rs5743708 were associated with TB in different populations [22–26], with inconsistent results. Xue et al. demonstrated that rs3804099 and rs3804100 were associated with pulmonary TB in the Tibetan population [24]. Also, another similar study with regard to Chinese Han found the two SNPs were not related to TB [16]. Our result was the same as the latter study. In addition, we validated the recent findings by Sánchez et al. in terms of no association between rs5743708 and TB [26].

The TLR4 is important in the activation of nuclear factor-κB by signal transduction related to different intracellular signaling systems and some inflammatory cytokines levels [27,28]. In addition, the TLR4 signaling pathway is known to maintain the balance between necrotic and apoptotic cell death caused by macrophages infected with *M. TB* [29]*.* Previous data have suggested that TLR4 mutant mice were more likely to be infected with *M. TB* [30]. In our research, the rs11536889 showed the strongest association with TB in the Tibetan population but not in the Chinese Han population, in agreement with another study aimed to detect such an SNP and TB in the Tibetan population [31] with the same CC+GC protective genotype. It was suggested that rs11536889 was a functional SNP, and it can influence not only the translational regulation of TLR4 expression and the post-transcriptional regulation [32]. Furthermore, this functional polymorphism was found to be associated with many infections and diseases, including rheumatoid arthritis [33], periodontitis [34] and sepsis [31].

TOLLIP has been previously described as a regulator of the interleukin-1 receptor pathway [35]. It can also directly interact with TLR2 and induce the phosphorylation and kinase activity of IRAK1 and thus stimulates termination of TLR2 signaling [36]. Interestingly, *TOLLIP* polymorphisms and its interaction with TLR2 and TLR4 in human monocytes were related to TB susceptibility [6]. *TOLLIP* rs3750920 is encoded on chromosome 11p15.5 and located in the fourth exon. Shah et al. demonstrated that the minor homozygote TT of rs3750920 was significantly associated with elevated mRNA expression, resulting in decreased risk against TB [6]. Consistent with this functional genotype, our study showed that TT genotype, compared with CC+CC, was associated with TB protection in the Tibetan population. However, we did not find this association in the initial study. A similar study conducted in Kampala, Uganda, gave results that showed significance between the risk of TB and *TOLLIP* rs5743867 polymorphism. We did not validate these findings in both Chinese Han and Tibetan populations. Although our study design was similar to the Shah et al., collecting patients’ peripheral blood, using a case–control study method, our result regarding rs5743899 was inconsistent with Shah et al. who found that this locus was associated with TB susceptibility. The discrepancies between the aforementioned results may be attributed to the different sample sizes and ethnicities [37]. Our results were also differing from our previous study [38], which demonstrated that polymorphisms in *TOLLIP* affected the risk of pulmonary TB. The different results may be due to the different study designs.

## Conclusion

In conclusion, our results demonstrated that the polymorphism of rs1898830 in *TLR2* was associated with TB susceptibility in both Chinese Han and Tibetan populations. Also, we found associations between rs11536889 in *TLR4* and rs3750920 in *TOLLIP* with TB in the Tibetan population, but not in the Han population. Further studies are needed to reveal the potential mechanisms of the involvement of these genes in the development of TB.

## Abbreviations

CI: confidence interval
HWE: Hardy–Weinberg equilibrium
iMLDR: improved multiplex ligase detection reaction
LD: linkage disequilibrium
M. TB: *Mycobacterium tuberculosis*
OR: odds ratio
SNP: single nucleotide polymorphism
TB: tuberculosis
TLR: toll-like receptor
TOLLIP: toll interacting protein

## References

[1] World Health Organization (2019) Global tuberculosis report 2019. https://www.who.int/tb/publications/global_report/en/

[2] Kramnik I., Dietrich W.F., Demant P. and Bloom B.R. (2000) Genetic control of resistance to experimental infection with virulent Mycobacterium tuberculosis. Proc. Natl. Acad. Sci. U.S.A. 97, 8560–8565 10.1073/pnas.150227197 10890913 PMC26987

[3] Misch E.A. and Hawn T.R. (2008) Toll-like receptor polymorphisms and susceptibility to human disease. Clin. Sci. (Lond.) 114, 347–360 10.1042/CS20070214 18230059

[4] Korbel D.S., Schneider B.E. and Schaible U.E. (2008) Innate immunity in tuberculosis: myths and truth. Microbes Infect. 10, 995–1004 10.1016/j.micinf.2008.07.039 18762264

[5] Kagan J.C. and Medzhitov R. (2006) Phosphoinositide-mediated adaptor recruitment controls Toll-like receptor signaling. Cell 125, 943–955 10.1016/j.cell.2006.03.047 16751103

[6] Shah J.A., Vary J.C., Chau T.T., Bang N.D., Yen N.T., Farrar J.J. et al. (2012) Human TOLLIP regulates TLR2 and TLR4 signaling and its polymorphisms are associated with susceptibility to tuberculosis. J. Immunol. 189, 1737–1746 10.4049/jimmunol.1103541 22778396 PMC3428135

[7] He S., Liang Y., Shao F. and Wang X. (2011) Toll-like receptors activate programmed necrosis in macrophages through a receptor-interacting kinase-3-mediated pathway. Proc. Natl. Acad. Sci. U.S.A. 108, 20054–20059 10.1073/pnas.1116302108 22123964 PMC3250173

[8] Fujiwara N., Porcelli S.A., Naka T., Yano I., Maeda S., Kuwata H. et al. (2013) Bacterial sphingophospholipids containing non-hydroxy fatty acid activate murine macrophages via Toll-like receptor 4 and stimulate bacterial clearance. Biochim. Biophys. Acta 1831, 1177–1184 10.1016/j.bbalip.2013.03.008 23545566

[9] Wu L., Hu Y., Li D., Jiang W. and Xu B. (2015) Screening toll-like receptor markers to predict latent tuberculosis infection and subsequent tuberculosis disease in a Chinese population. BMC Med. Genet. 16, 19 10.1186/s12881-015-0166-1 25928077 PMC4421918

[10] Arji N., Busson M., Iraqi G., Bourkadi J.E., Benjouad A., Bouayad A. et al. (2014) Genetic diversity of TLR2, TLR4, and VDR loci and pulmonary tuberculosis in Moroccan patients. J. Infect. Dev. Countr. 8, 430–440 10.3855/jidc.3820 24727508

[11] Wang Y., Zhang M.M., Huang W.W., Wu S.Q., Wang M.G., Tang X.Y. et al. (2018) Polymorphisms in Toll-like receptor 10 and tuberculosis susceptibility: evidence from three independent series. Front. Immunol. 9, 309 10.3389/fimmu.2018.00309 29527210 PMC5829065

[12] Zhang M., Tang X., Wang Y., Wu S., Wang M., Liu Q. et al. (2018) Variants of TLR1 associated with tuberculosis susceptibility in the Chinese Tibetan population but not in Han Chinese. Infect. Genet. Evol. 61, 53–59 10.1016/j.meegid.2018.02.021 29454979

[13] Etokebe G.E., Skjeldal F., Nilsen N., Rodionov D., Knezevic J., Bulat-Kardum L. et al. (2010) Toll-like receptor 2 (P631H) mutant impairs membrane internalization and is a dominant negative allele. Scand. J. Immunol. 71, 369–381 10.1111/j.1365-3083.2010.02379.x 20500688

[14] Du W., Cheng J., Ding H., Jiang Z., Guo Y. and Yuan H. (2014) A rapid method for simultaneous multi-gene mutation screening in children with nonsyndromic hearing loss. Genomics 104, 264–270 10.1016/j.ygeno.2014.07.009 25149764

[15] Mittal M., Biswas S.K., Singh V., Arela N., Katoch V.M., Das R. et al. (2018) Association of Toll like receptor 2 and 9 gene variants with pulmonary tuberculosis: exploration in a northern Indian population. Mol. Biol. Rep. 45, 469–476 10.1007/s11033-018-4182-z 29675696

[16] Zhang J., Zhao Z., Zhong H., Wu L., Zhou W., Peng W. et al. (2018) Importance of common TLR2 genetic variants on clinical phenotypes and risk in tuberculosis disease in a Western Chinese population. Infect. Genet. Evol. 60, 173–180 10.1016/j.meegid.2018.02.031 29486365

[17] Underhill D.M., Ozinsky A., Hajjar A.M., Stevens A., Wilson C.B., Bassetti M. et al. (1999) The Toll-like receptor 2 is recruited to macrophage phagosomes and discriminates between pathogens. Nature 401, 811–815 10.1038/44605 10548109

[18] Lorenz E., Mira J.P., Cornish K.L., Arbour N.C. and Schwartz D.A. (2000) A novel polymorphism in the toll-like receptor 2 gene and its potential association with staphylococcal infection. Infect. Immun. 68, 6398–6401 10.1128/IAI.68.11.6398-6401.2000 11035751 PMC97725

[19] Tomiyama R., Meguro A., Ota M., Katsuyama Y., Nishide T., Uemoto R. et al. (2009) Investigation of the association between Toll-like receptor 2 gene polymorphisms and Behcet’s disease in Japanese patients. Hum. Immunol. 70, 41–44 10.1016/j.humimm.2008.10.014 19014987

[20] Alvarez A.E., Marson F.A.L., Bertuzzo C.S., Bastos J.C.S., Baracat E.C.E., Brandao M.B. et al. (2018) Association between single nucleotide polymorphisms in TLR4, TLR2, TLR9, VDR, NOS2 and CCL5 genes with acute viral bronchiolitis. Gene 645, 7–17 10.1016/j.gene.2017.12.022 29253610 PMC7127094

[21] Rädler D. (2014) Mechanisms of immune regulation during development of atopic diseases in childhood. *Ludwig-Maximilians-Universität München*

[22] Zhao Y., Bu H., Hong K., Yin H., Zou Y.L., Geng S.J. et al. (2015) Genetic polymorphisms of CCL1 rs2072069 G/A and TLR2 rs3804099 T/C in pulmonary or meningeal tuberculosis patients. Int. J. Clin. Exp. Pathol. 8, 12608–12620 26722451 PMC4680396

[23] Schurz H., Daya M., Moller M., Hoal E.G. and Salie M. (2015) TLR1, 2, 4, 6 and 9 variants associated with tuberculosis susceptibility: a systematic review and meta-analysis. PLoS ONE 10, e0139711 10.1371/journal.pone.0139711 26430737 PMC4592262

[24] Xue X., Qiu Y., Jiang D., Jin T., Yan M., Zhu X. et al. (2017) The association analysis of TLR2 and TLR4 gene with tuberculosis in the Tibetan Chinese population. Oncotarget 8, 113082–9 10.18632/oncotarget.22996 29348888 PMC5762573

[25] Guo X.G. and Xia Y. (2015) The rs5743708 gene polymorphism in the TLR2 gene contributes to the risk of tuberculosis disease. Int. J. Clin. Exp. Pathol. 8, 11921–11928 26617949 PMC4637765

[26] Sanchez D., Lefebvre C., Rioux J., Garcia L.F. and Barrera L.F. (2012) Evaluation of Toll-like receptor and adaptor molecule polymorphisms for susceptibility to tuberculosis in a Colombian population. Int. J. Immunogenet. 39, 216–223 10.1111/j.1744-313X.2011.01077.x 22221660

[27] Vogel S.N. and Fenton M. (2003) Toll-like receptor 4 signalling: new perspectives on a complex signal-transduction problem. Biochem. Soc. Trans. 31, 664–668 10.1042/bst0310664 12773178

[28] Kawai T. and Akira S. (2006) TLR signaling. Cell Death Differ. 13, 816–825 10.1038/sj.cdd.4401850 16410796

[29] Sanchez D., Rojas M., Hernandez I., Radzioch D., Garcia L.F. and Barrera L.F. (2010) Role of TLR2- and TLR4-mediated signaling in Mycobacterium tuberculosis-induced macrophage death. Cell. Immunol. 260, 128–136 10.1016/j.cellimm.2009.10.007 19919859

[30] Branger J., Leemans J.C., Florquin S., Weijer S., Speelman P. and Van Der Poll T. (2004) Toll-like receptor 4 plays a protective role in pulmonary tuberculosis in mice. Int. Immunol. 16, 509–516 10.1093/intimm/dxh052 14978024

[31] Wang H., Wei Y., Zeng Y., Qin Y., Xiong B., Qin G. et al. (2014) The association of polymorphisms of TLR4 and CD14 genes with susceptibility to sepsis in a Chinese population. BMC Med. Genet. 15, 123 10.1186/s12881-014-0123-4 25394369 PMC4411696

[32] Sato K., Yoshimura A., Kaneko T., Ukai T., Ozaki Y., Nakamura H. et al. (2012) A single nucleotide polymorphism in 3′-untranslated region contributes to the regulation of Toll-like receptor 4 translation. J. Biol. Chem. 287, 25163–25172 10.1074/jbc.M111.338426 22661708 PMC3408206

[33] Yang H., Wei C., Li Q., Shou T., Yang Y., Xiao C. et al. (2013) Association of TLR4 gene non-missense single nucleotide polymorphisms with rheumatoid arthritis in Chinese Han population. Rheumatol. Int. 33, 1283–1288 10.1007/s00296-012-2536-8 23129427

[34] Fukusaki T., Ohara N., Hara Y., Yoshimura A. and Yoshiura K. (2007) Evidence for association between a Toll-like receptor 4 gene polymorphism and moderate/severe periodontitis in the Japanese population. J. Periodontal Res. 42, 541–545 10.1111/j.1600-0765.2007.00979.x 17956467

[35] Burns K., Clatworthy J., Martin L., Martinon F., Plumpton C., Maschera B. et al. (2000) Tollip, a new component of the IL-1RI pathway, links IRAK to the IL-1 receptor. Nat. Cell Biol. 2, 346–351 10.1038/35014038 10854325

[36] Zhang G. and Ghosh S. (2002) Negative regulation of toll-like receptor-mediated signaling by Tollip. J. Biol. Chem. 277, 7059–7065 10.1074/jbc.M109537200 11751856

[37] Wang M., Xu G., Lu L., Xu K., Chen Y., Pan H. et al. (2016) Genetic polymorphisms of IL-17A, IL-17F, TLR4 and miR-146a in association with the risk of pulmonary tuberculosis. Sci. Rep. 6, 28586 10.1038/srep28586 27339100 PMC4919632

[38] Wu S., Huang W., Wang D., Wang Y., Wang M., Zhang M. et al. (2018) Evaluation of TLR 2, TLR 4, and TOLLIP polymorphisms for their role in tuberculosis susceptibility. APMIS 126, 501–508 10.1111/apm.12855 29924447

